# Efficacy and safety of dapagliflozin in children with kidney disease: real-world data

**DOI:** 10.1007/s00467-024-06481-8

**Published:** 2024-08-06

**Authors:** Naye Choi, Ji Hyun Kim, Peong Gang Park, Hyeonju Lee, Jeesu Min, Hye Won Park, Yo Han Ahn, Hee Gyung Kang

**Affiliations:** 1 Department of Pediatrics, Korea University Anam Hospital, Seoul, Republic of Korea; 2 Department of Pediatrics, Seoul National University College of Medicine, Seoul, Republic of Korea; 3 Department of Pediatrics, Seoul National University Bundang Hospital, Seongnam, Republic of Korea; 4 Department of Pediatrics, Ajou University School of Medicine, Suwon, Republic of Korea; 5 Department of Pediatrics, Seoul National University Children’s Hospital, Seoul, Republic of Korea; 6 Department of Pediatrics, Chungnam National University Sejong Hospital, Sejong, Republic of Korea; 7 Suwon Center for Environmental Disease Atopy, Ajou University Hospital, Suwon, Republic of Korea

**Keywords:** Sodium-glucose transporter 2 inhibitors, Chronic kidney disease, Proteinuria

## Abstract

**Background:**

Dapagliflozin, a sodium-glucose cotransporter-2 inhibitor, has shown results in slowing estimated glomerular filtration rate (eGFR) decline and reducing proteinuria in adult patients with chronic kidney disease. This retrospective study examines dapagliflozin’s effects in 22 children with kidney disease and proteinuria.

**Methods:**

Children with a median age of 15.6 years were treated with dapagliflozin for > 3 months between July 2022 and December 2023. All children had been treated with either an angiotensin-converting enzyme inhibitor or angiotensin receptor blocker for at least 1 month before starting dapagliflozin.

**Results:**

The most common kidney disease diagnoses in this study included Alport syndrome (*n* = 7) and medication-resistant nephrotic syndrome or focal segmental glomerulosclerosis (*n* = 7). After 6.1 months of treatment, dapagliflozin treatment did not result in significant changes in eGFR or proteinuria. However, at the latest follow-up, a statistically significant decrease in eGFR was noted (65.5 compared to the baseline 71.1 mL/min/1.73 m^2^, *P* = 0.003). Proteinuria remained stable between baseline and the last follow-up (final spot urine protein/creatinine ratio (uPCR) 0.7 vs. baseline uPCR 0.6 mg/mg, *P* = 0.489). In the subgroup analysis of children treated for > 8 months, the eGFR decline post-treatment changed from − 0.5 to − 0.2 ml/min/1.73 m^2^ per month (*P* = 0.634). Only two children discontinued dapagliflozin due to suspected adverse events.

**Conclusions:**

Dapagliflozin has not been associated with serious side effects. Further prospective clinical trials are needed to confirm the efficacy and safety of dapagliflozin in children with kidney disease.

**Graphical abstract:**

**Figure:**
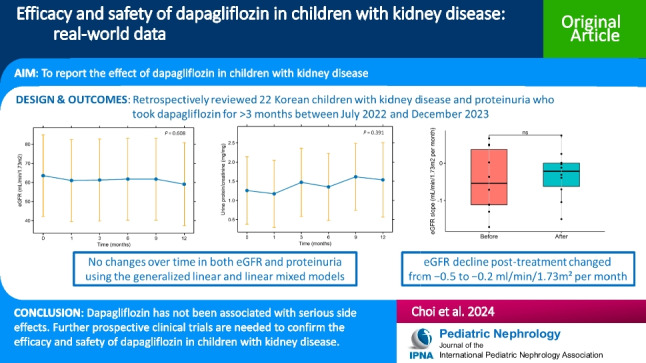
A higher resolution version of the Graphical abstract is available as Supplementary information

**Supplementary Information:**

The online version contains supplementary material available at 10.1007/s00467-024-06481-8.

## Introduction

Chronic kidney disease (CKD) affects adults and children, contributing to significant social costs [1, 2]. CKD in children is primarily caused by congenital anomalies of the kidneys and urinary tract (CAKUT), followed by glomerulopathies [3]. However, treating the underlying disease is often challenging in many practical situations; therefore, as in adults, second-line treatment strategies for children with CKD focus on controlling hypertension and proteinuria through renin–angiotensin–aldosterone system inhibition to preserve kidney function as long as possible. In adults, other classes of medications such as spherical carbon adsorbents, mineralocorticoid receptor antagonists, and sodium–glucose cotransporter 2 (SGLT2) inhibitors are approved to slow down CKD progression in one or more countries; however, none are indicated for children to date [4–6].

Dapagliflozin and empagliflozin are SGLT2 inhibitors approved for CKD. In addition to lowering blood sugar by inhibiting SGLT2s in the proximal tubules of the kidneys, these medications have been shown to reduce the decline of estimated glomerular filtration rate (eGFR) in adult patients with CKD, regardless of the severity, most probably by relieving the hyperfiltration of the remnant glomeruli in CKD [7–10]. While the Kidney Disease: Improving Global Outcomes (KDIGO) guidelines for the treatment of diabetes in patients with CKD recommend SGLT2 inhibitors as the first-line therapy [11], the KDIGO guideline for glomerular disease management, published in 2021, did not recommend the utilization of SGLT2 inhibitors in IgA nephropathy (IgAN) without diabetes [12]. However, recently published results of clinical trials revealed that the therapeutic potential of SGLT2 inhibitors extends to patients with IgAN [13] and focal segmental glomerulosclerosis (FSGS) with reduced proteinuria and eGFR decline [8, 14]. Since IgAN and FSGS are common causes of pediatric CKD of glomerular origin, pediatric nephrologists believe that SGLT2 inhibitors could potentially be beneficial for the pediatric CKD population [15, 16]. However, trials assessing the efficacy and safety of SGLT2 inhibitors in CKD did not include children. To date, two published studies from one center examined the efficacy of dapagliflozin in children with CKD with a median age of 10.4 years [17, 18]. Conversely, previous studies have confirmed the safety of dapagliflozin on type 2 diabetes and heart failure in the pediatric population, and empagliflozin is approved for children with diabetes aged ≥ 10 years in the United States [19–21].

In South Korea, dapagliflozin is approved for use in adults with CKD. It had been briefly prescribed for off-label use for the treatment of proteinuria in children with CKD at our institution upon request of pediatric nephrologists before the Korean Food and Drug Administration decided to ban this medication based on the lack of supporting evidence in children. Here, we report our experience of the real-world data.

## Materials and methods

### Patients and dapagliflozin treatment

Dapagliflozin was prescribed for children aged < 19 years with kidney disease and proteinuria despite taking an angiotensin-converting-enzyme inhibitor (ACEi) or angiotensin receptor blocker (ARB), with an eGFR of ≥ 20 ml/min/1.73 m^2^ at baseline between July 2022 and December 2023. Clinicians adjusted the dose based on body weight and eGFR, but there was no standardized protocol. All eligible patients who consented received dapagliflozin during this period. Children treated with dapagliflozin for > 3 months were reviewed in this study. None of the children had any of the following pre-existing conditions: polycystic kidney disease, type 1 diabetes, uncontrolled urinary tract infections, kidney transplantation, current cancer diagnosis, and evidence of liver disease. Proteinuria was defined as a spot urine protein/creatinine ratio (uPCR) of ≥ 0.2 mg/mg. The term “eGFR dipping” refers to the immediate reduction in eGFR instantly after starting the dapagliflozin treatment. Decreased proteinuria was defined as a uPCR lower than the initial treatment level at the last visit, and glycosuria as the presence of glucose 4 + in a patient’s spot urine stick test at the 1-month visit. This retrospective study was approved by the Seoul National University Hospital institutional review board (IRB no. 2304–049-1421).

### Clinical outcomes

Data on the clinical characteristics and laboratory data of children were obtained. For eGFR calculations, the CKiD Under 25 (U25) GFR estimating equations were used [22]. The study’s efficacy outcomes included reduced proteinuria, eGFR changes, and eGFR decline. The eGFR decline during dapagliflozin treatment (eGFR at the last visit SGLT2i therapy − eGFR at the initial SGLT2i therapy) / the treatment duration in months (X) was compared to the eGFR changes per month before the treatment initiation (eGFR at the initial SGLT2i therapy − eGFR at X months before SGLT2i therapy) / X.

For subgroup analysis, children were classified based on the underlying cause of kidney disease, obesity [body mass index (BMI) > 97 percentile], and CKD stages. Moreover, factors associated with the dipping and decreased proteinuria were analyzed.

### Statistical analyses

Data are expressed as numbers (percentages) for categorical variables and as medians with interquartile ranges (IQRs) for continuous variables. A generalized linear mixed model for non-normally distributed data and a linear mixed model for normally distributed data were employed. These models incorporated a random effect to account for time-related effects associated with longitudinal measurements and examined the eGFR and proteinuria changes during the dapagliflozin treatment. Differences in clinical outcomes before and after dapagliflozin treatments were analyzed using the paired t-test or Wilcoxon signed-rank sum test for continuous variables and the McNemar test for categorical variables. Statistical analysis was performed using Statistical Package for Social Sciences v. 27.0 (SPSS Inc. Chicago, IL, USA) and R software v. 4.3.0 (The Comprehensive R Archive Network, Vienna, Austria). The R package for ggpubr was used to create boxplots and line graphs. The lmerTest package uses the Satterthwaite method to approximate the degree of freedoms of the fixed effects in the linear mixed‐effect model. A *P*-value of < 0.05 was considered statistically significant for all tests.

## Results

### Patient characteristics

The clinical characteristics and medication history of children are summarized in Table 1 and Supplementary Table 1. Dapagliflozin was prescribed to a total of 22 children (M:F ratio, 12:10; median age, 15.6 years; baseline eGFR 71.1 ml/min/1.73 m^2^; uPCR 0.6 mg/mg) for 6.1 (IQR 4.0–9.6) months. Their diagnoses included Alport syndrome (*n* = 7), medication-resistant nephrotic syndrome or FSGS (*n* = 7, genetic in 3 and non-genetic in 4), IgAN or IgA vasculitis (*n* = 5), atypical hemolytic uremic syndrome (*n* = 2), and CAKUT (*n* = 1). Of the 22 children, 13 were on ACEi, 7 were taking ARB, and 2 received a combination of both ACEi and ARB.

**Table 1:** Clinical characteristics of the patients

Characteristic			
Sex (Male:Female)	12:10		
Age, years, median (IQR)	15.6 (12.9–17.2)		
Previous ACEi or ARB, n (%)			
ACEi	13 (59.1)		
ARB	7 (31.8)		
Both	2 (9.1)		
Dapagliflozin dose, n (%)			
5 mg	2 (9.1)		
10 mg	15 (68.2)		
5 mg → 10 mg	2 (9.1)		
10 mg → 5 mg	3 (13.6)		
Primary renal dianosis, n (%)			
Alport syndrome	7 (31.8)		
Medication-resistant nephrotic syndrome or FSGS	7 (31.8)		
IgAN or IgA vasculitis	5 (22.7)		
Atypical hemolytic uremic syndrome	2 (9.1)		
CAKUT	1 (4.5)		
Treatment duration, month, median (IQR)	6.1 (4.0–9.6)		
Clinical characteristics before and after treatment	At baseline	Last treatment	*P*-value
eGFR, ml/min/1.73 m^2^, median (IQR)	71.1 (39.4–93.9)	65.5 (33.1–92.7)	0.033
uPCR, mg/mg, median (IQR)	0.6 (0.4–1.5)	0.7 (0.3–1.7)	0.489
Albumin, g/dL, median (IQR)	4.3 (4.0–4.4)	4.3 (3.9–4.4)	0.214
Uric acid, mg/dL, median (IQR)	5.7 (5.0–6.3)	5.6 (3.8–6.6)	0.702
Potassium, mmol/L, median (IQR)	4.2 (3.9–4.4)	4.1 (4.1–4.8)	0.461
Body mass index, kg/m2, median (IQR)	19.5 (17.0–22.4)	19.4 (17.2–23.1)	0.159
Systolic blood pressure, mmHg, median (IQR)	113.0 (104.0–121.0)	112.0 (107.0–123.0)	0.233
Diastolic blood pressure, mmHg, median (IQR)	69.0 (63.0–74.0)	73.0 (71.0–80.0)	0.001
eGFR and uPCR change stratified by etiology	At baseline	Last treatment	*P*-value
Alport syndrome			
eGFR, ml/min/1.73 m^2^, median (IQR)	81.5 (38.0–102.4)	69.0 (33.7–102.6)	0.397
uPCR, mg/mg, median (IQR)	0.7 (0.5–2.5)	0.7 (0.5–0.9)	0.248
Medication-resistant nephrotic syndrome or FSGS			
eGFR, ml/min/1.73 m^2^, median (IQR)	48.1 (24.1–68.5)	33.5 (24.4–84.2)	0.310
uPCR, mg/mg, median (IQR)	1.4 (0.5–2.7)	2.2 (1.3–3.8)	0.128
IgAN or IgAV			
eGFR, ml/min/1.73 m^2^, median (IQR)	92.7 (81.4–105.5)	88.9 (71.5–106.6)	0.345
uPCR, mg/mg, median (IQR)	0.3 (0.3–0.4)	0.4 (0.2–0.6)	0.465

*ACEi* angiotensin converting enzyme inhibitor, *ARB* angiotensin receptor blockers, *CAKUT* congenital anomaly of the kidney and urinary tract, *eGFR* estimated glomerular filtration rate *FSGS* focal segmental glomerulosclerosis, *IgAN* IgA nephropathy, *IgAV* IgA vasculitis, *IQR* interquartile ranges, *uPCR* urinary protein-to-creatinine ratio

### Clinical outcomes

**Fig. 1:**
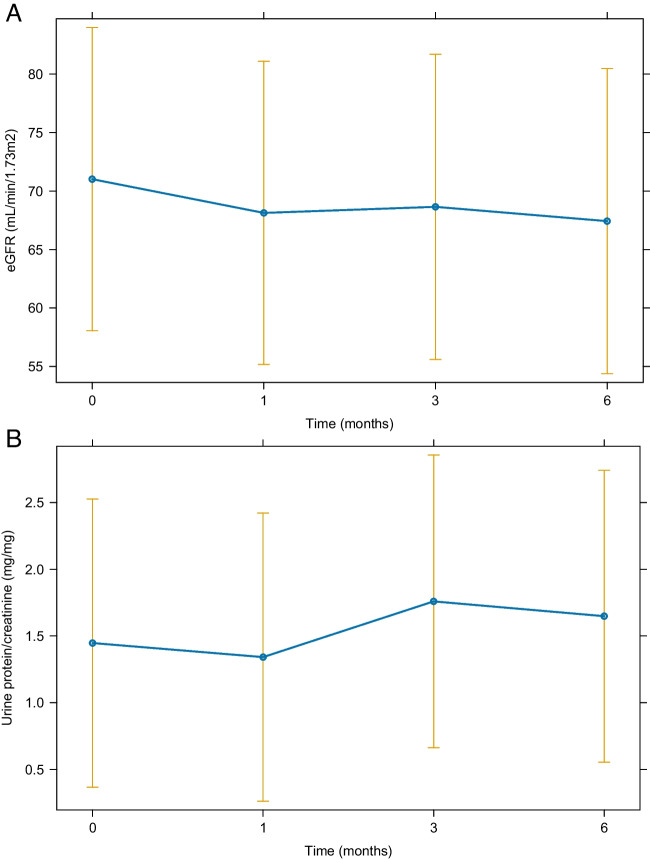
Changes in the estimated glomerular filtration rate (eGFR) (**A**) and proteinuria (**B**) after the dapagliflozin treatment. The estimated marginal means for each time point are presented, and vertical bars represent the 95% confidence interval

**Fig. 2:**
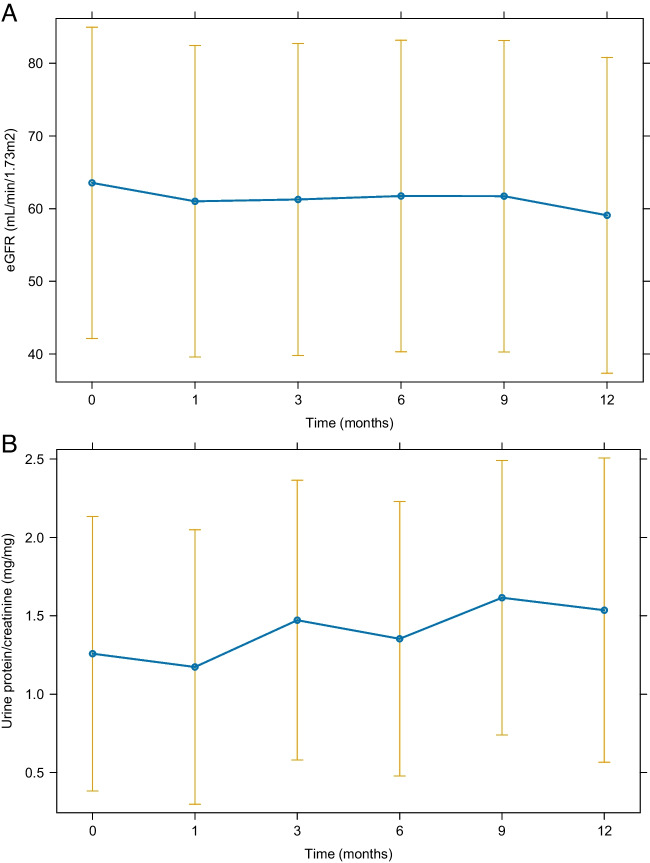
Changes in the estimated glomerular filtration rate (eGFR) (**A**) and proteinuria (**B**) for patients treated > 8 months. The estimated marginal means for each time point are presented, and vertical bars represent the 95% confidence interval

**Fig. 3:**
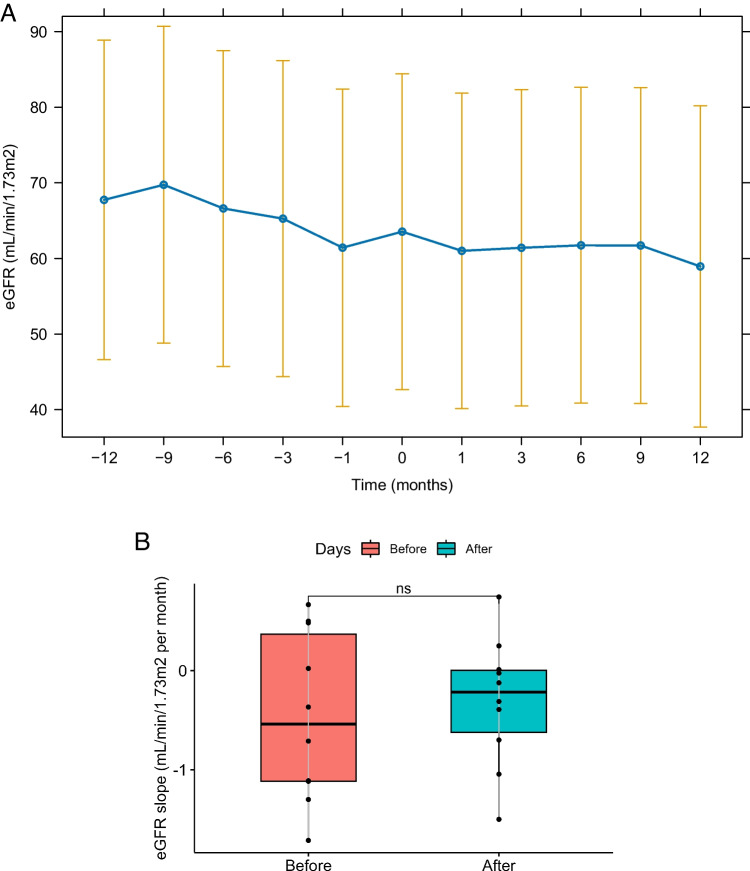
**A** Changes in the estimated glomerular filtration rate (eGFR) before and after dapagliflozin treatment for patients treated > 8 months. The estimated marginal means for each time point are presented, and vertical bars represent the 95% confidence interval. **B** eGFR slope before and after dapagliflozin treatment for patients treated > 8 months. In the box and whisker plots, lines within boxes represent median values; the top and bottom lines of the boxes represent the 75th and 25th percentiles, respectively. ns, not significant

The baseline eGFR was 71.1 (IQR 39.4–93.9) ml/min/1.73 m^2^, and the latest follow-up eGFR was 65.5 (IQR 33.1–92.7) ml/min/1.73 m^2^ after dapagliflozin treatment, with a *P*-value of 0.033. There was no significant difference between the final uPCR of 0.7 (IQR 0.3–1.7) mg/mg and the baseline uPCR of 0.6 (IQR 0.4–1.5) mg/mg at the last follow-up (*P* = 0.489). Other serum chemistry values, BMI, and systolic blood pressure at the last follow-up did not also significantly change from those at baseline except for diastolic blood pressure (Table 1 and Supplementary Table 1). In the subgroup of 10 children treated for > 8 months (median, 9.7 months; IQR, 8.5–11.5 months), the baseline eGFR was 51.2 (IQR 33.4–93.9) ml/min/1.73 m^2^ and the eGFR at the last follow-up was 42.9 (IQR 28.9–90.5) ml/min/1.73 m^2^ (*P* = 0.037). The uPCR at baseline was 0.9 mg/mg and at the last follow-up was 0.8 mg/mg (*P* = 0.441). However, further analysis using the generalized linear and linear mixed models revealed no changes over time in both eGFR and uPCR (Fig. 1 and Fig. 2). The eGFR decline changed from − 0.5 ml/min/1.73 m^2^ per month (IQR − 1.2 to 0.5) before the dapagliflozin treatment to − 0.2 ml/min/1.73 m^2^ per month (IQR, − 0.8 to 0.1) during the dapagliflozin treatment (Fig. 3(B)), although not statistically significant (*P* = 0.634). The subgroup analysis revealed that none of the underlying disease groups (Figure S1), obesity (Figure S2), or CKD stages (Figure S3) showed statistically significant eGFR or proteinuria changes with dapagliflozin, whereas eGFR seemed to improve in the obese group compared with the nonobese group (Figure S2(A)).

The eGFR dipped in 14 children, especially in the Alport group and medication-resistant nephrotic syndrome group (Figure S1(A)) and in CKD G1 and G2 (Figure S3(A)). However, none of the factors such as age at the treatment initiation, baseline eGFR, treatment duration, initial BMI, and initial blood pressure were associated with eGFR dipping. The amount of proteinuria decreased in 10 children treated with dapagliflozin; however, associated factors could not be found. Glycosuria was observed in 10 children with higher initial eGFR levels and lower initial proteinuria compared with the no glycosuria group: eGFR, 84.2 (IQR 62.5–121.6) vs. 46.9 (IQR 35.3–89.1) ml/min/1.73 m^2^ (*P* = 0.043) and uPCR, 0.4 (IQR 0.3–0.6) vs. 1.1 (IQR 0.5–2.3) mg/mg (*P* = 0.014).

### Adverse events

Dapagliflozin treatment was discontinued in two children due to suspected adverse events: rhabdomyolysis (*n* = 1) and alopecia (*n* = 1). The patient with rhabdomyolysis presented with pain in the right calf and thigh, with initial laboratory findings showing myoglobin levels of 3010.3 ng/mL and creatinine kinase (CK) levels of 2630 IU/L. The patient was taking ezetimibe, which was discontinued, and hydration therapy was initiated. Three days later, myoglobin levels decreased to 528.9 ng/mL and CK levels to 1085 IU/L. However, the relevance of these events with the medication was unclear. Two other children opted out from dapagliflozin treatment due to eGFR decline of < 20 ml/min/1.73 m^2^. No children reported urinary tract infection, hypoglycemia, hypovolemia, or hypotension.

## Discussion

The mean duration of 6.1 months of dapagliflozin treatment decreased the eGFR but did not increase the proteinuria in children with kidney disease. However, the eGFR decline seemed to improve with dapagliflozin in children treated for > 8 months, and no serious adverse events occurred.

In previous studies on 23 children with hereditary kidney disease with proteinuria [17, 18], dapagliflozin treatment for 24 weeks did not show efficacy. An adult study of a 6-week treatment with 58 patients with proteinuria also failed to show any benefit [23]. As the kidney function of hereditary kidney diseases such as Alport syndrome often deteriorates rapidly with aggravated proteinuria during adolescence [24, 25], it is challenging to determine whether the observed eGFR decline in this study is primarily due to the natural progression of the disease or the effect of SGLT2 inhibitors. To overcome this limitation, we compared the eGFR slope before and after dapagliflozin treatment. The study of empagliflozin suggests that the eGFR slope offers a more accurate assessment of SGLT2 inhibitor efficacy [8]. Furthermore, kidney protection by decelerating CKD progression with SGLT2 inhibition, based on the analysis of eGFR slope data, was more evident in the early CKD stage, irrespective of albuminuria [8]. Therefore, a well-designed study is needed to determine the effect of SGLT2 inhibitors in children.

In the subgroup analysis in this study, dipping was noted in the Alport group, medication-resistant nephrotic syndrome group, CKD G1, and CKD G2. The initial acute reduction in eGFR following dapagliflozin treatment could potentially be associated with the reduction in albuminuria in patients with CKD [26]. Since eGFR dipping was noted in CKD G1 and G2 as in a previous report [27] and those whose proteinuria was reduced with dapagliflozin had higher initial eGFR, the more pronounced eGFR dipping may suggest increased effectiveness of dapagliflozin against proteinuria, especially in children with early-stage CKD. However, such correlation could not be established, most probably because of the small number of children and the short treatment duration. Similarly, glucosuria was observed in children with better kidney function and milder proteinuria in this study, implying that SGLT2 inhibitors might work better for early-stage CKD [28]. Therefore, future studies should stratify by etiology and CKD stage in children to identify those who may benefit from this medication.

No serious adverse effects of dapagliflozin were reported in this study, like a recent review article reporting that all children treated with dapagliflozin did not complain of common adverse reactions, such as urinary tract infection, vomiting, hypoglycemia, and diabetic ketoacidosis [16, 21]. However, a study involving 38 children with heart failure and a median age of 12.2 years reported that six children experienced symptomatic urinary tract infections requiring antibiotic treatment; however, no instances of symptomatic hypoglycemia or hypovolemia were reported [10]. The uncertainty surrounding the use of SGLT2 inhibitors in CAKUT patients is linked to the increased risk of urinary tract infections. However, no urinary tract infection events occurred during dapagliflozin treatment in the CAKUT patient in this study. Further, dehydration, hypovolemia, and hypotension may occur and should be monitored, especially in children with CAKUT or polyuria, who might be more vulnerable than others [15]. Therefore, the benefits of dapagliflozin in children with kidney disease are assumed to surpass the potential risks.

A limitation of this study is the relatively short treatment duration, as SGLT2 inhibitors typically require 1–2 years to obtain an effect on GFR. Furthermore, the small sample size presents challenges in the interpretation of results and may lead to underpowered findings for relevant outcomes. The absence of a comparison group in this study is another notable limitation. Due to the heterogeneity of the study group, meaningful and conclusive data interpretation could not be drawn. Moreover, proteinuria was considered an indicator in this study, whereas other studies in adult populations have used albuminuria, a more direct indicator of glomerular hyperfiltration/strain. Nevertheless, this study holds significance due to the scarcity of reported studies on dapagliflozin treatment in children with CKD.

In conclusion, dapagliflozin has not been linked with serious side effects; however, long-term investigations are needed to fully understand its efficacy and potential adverse effects in children.

## Supplementary information

Below is the link to the electronic supplementary material.

Graphical abstract (PPTX 196 KB)

Supplementary Material 1.

Supplementary Material 2.

Supplementary Material 3.

Supplementary Material 4.

## Data Availability

The data underlying this article will be shared on reasonable request to the corresponding author.
