# Supplementary material for: Efficacy and safety of dapagliflozin in children with kidney disease: real-world data
Source: Pediatr Nephrol. 2024 Aug 6;39(12):3551–8. doi: 10.1007/s00467-024-06481-8 (PMC11511754; doi:10.1007/s00467-024-06481-8)
Supplement: Supplementary file 1 — Graphical abstract (PPTX 196 KB) [file 467_2024_6481_MOESM1_ESM.pptx]

## Slide 1
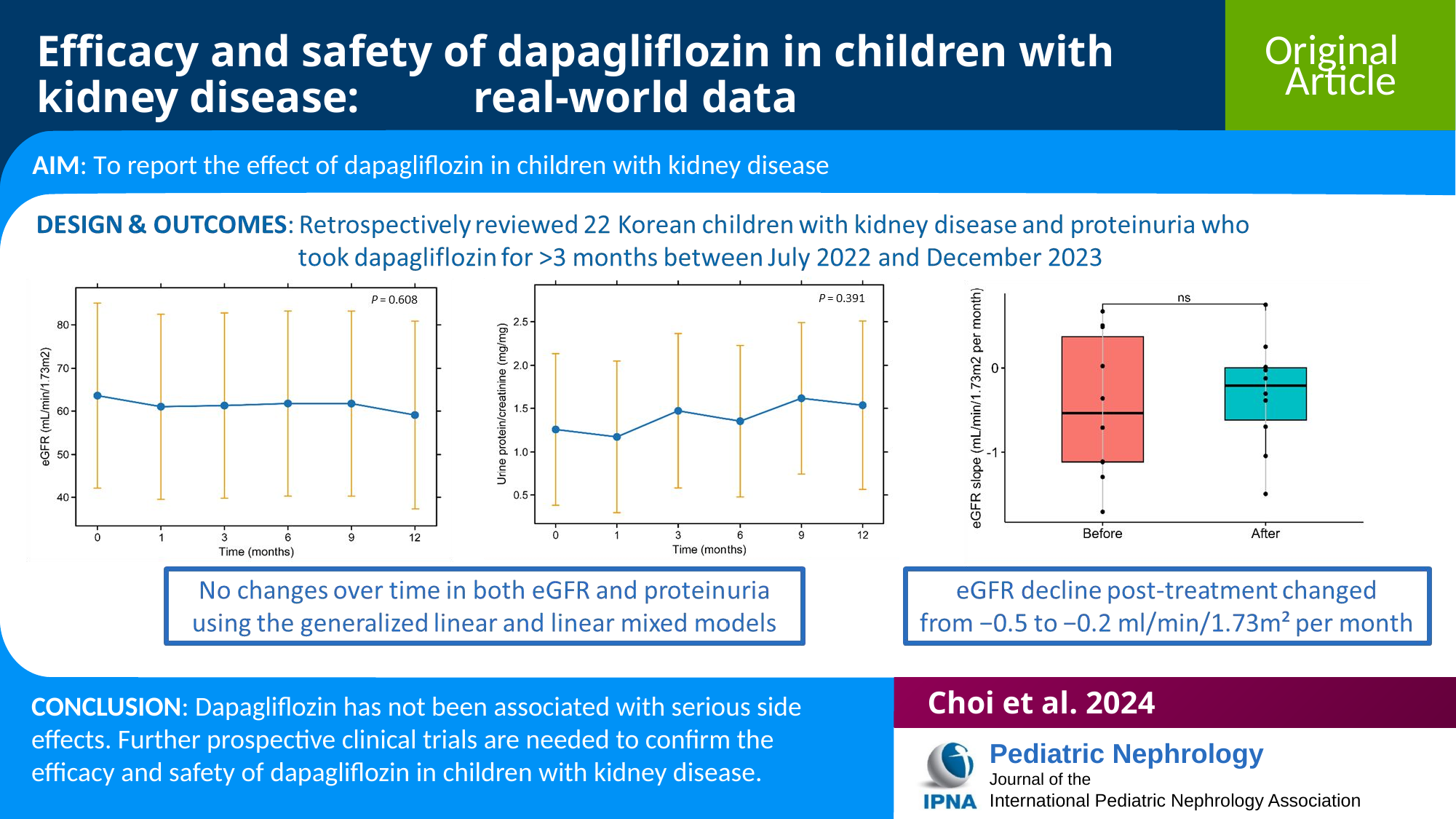

Efficacy and safety of dapagliflozin in children with kidney disease: 	real-world data
AIM: To report the effect of dapagliflozin in children with kidney disease
Choi et al. 2024
CONCLUSION: Dapagliflozin has not been associated with serious side effects. Further prospective clinical trials are needed to confirm the efficacy and safety of dapagliflozin in children with kidney disease.
