# Supplementary figures and images for: Efficacy and safety of dapagliflozin in children with kidney disease: real-world data
Source: Pediatr Nephrol. 2024 Aug 6;39(12):3551–8. doi: 10.1007/s00467-024-06481-8 (PMC11511754; doi:10.1007/s00467-024-06481-8)

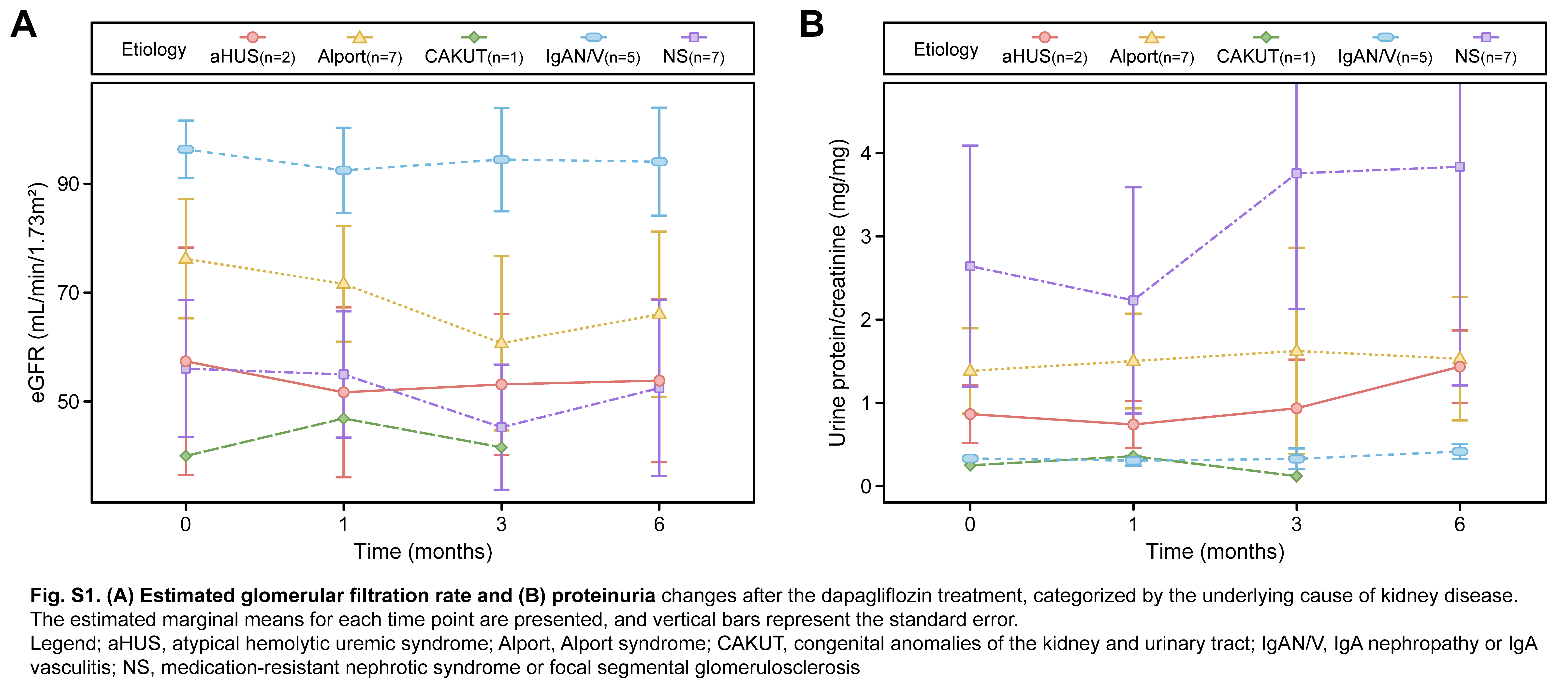

Supplement: Supplementary file 3 — Supplementary Material 2. [file 467_2024_6481_MOESM3_ESM.tif]

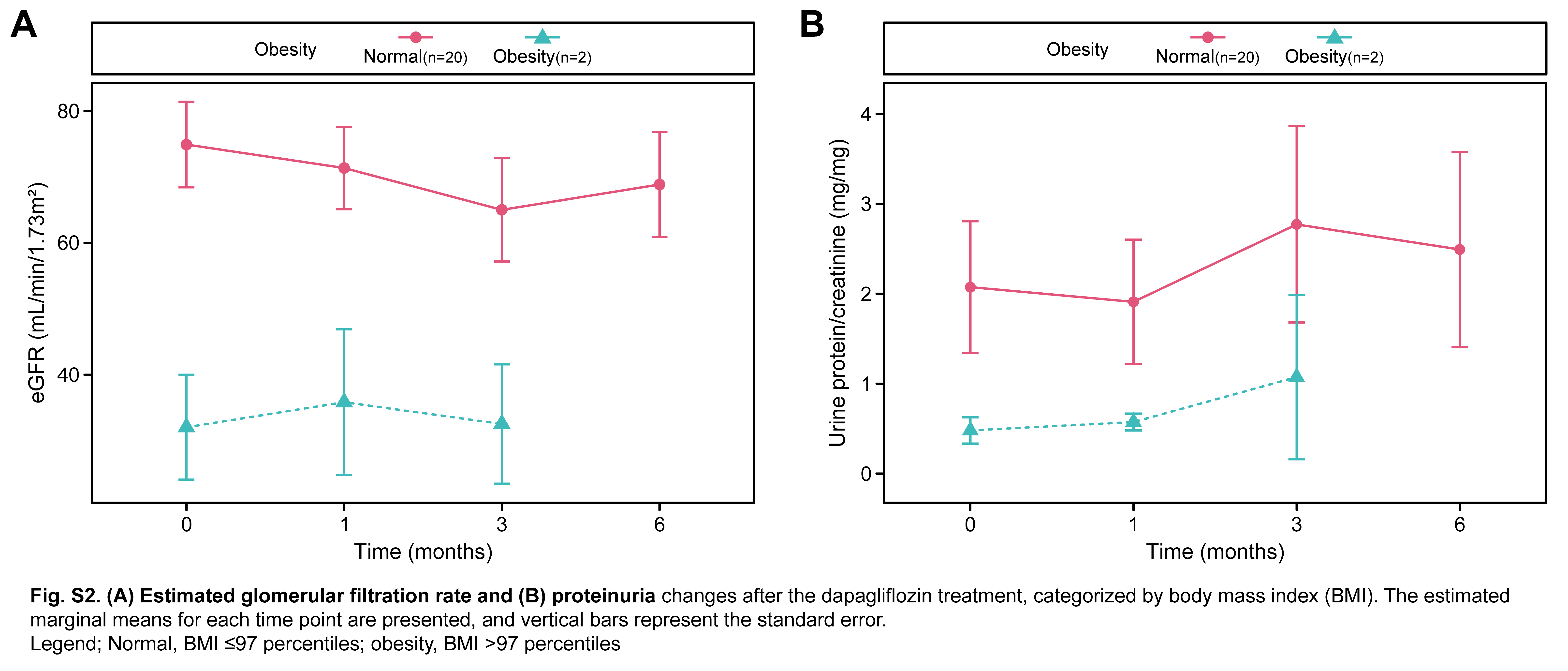

Supplement: Supplementary file 4 — Supplementary Material 3. [file 467_2024_6481_MOESM4_ESM.tif]

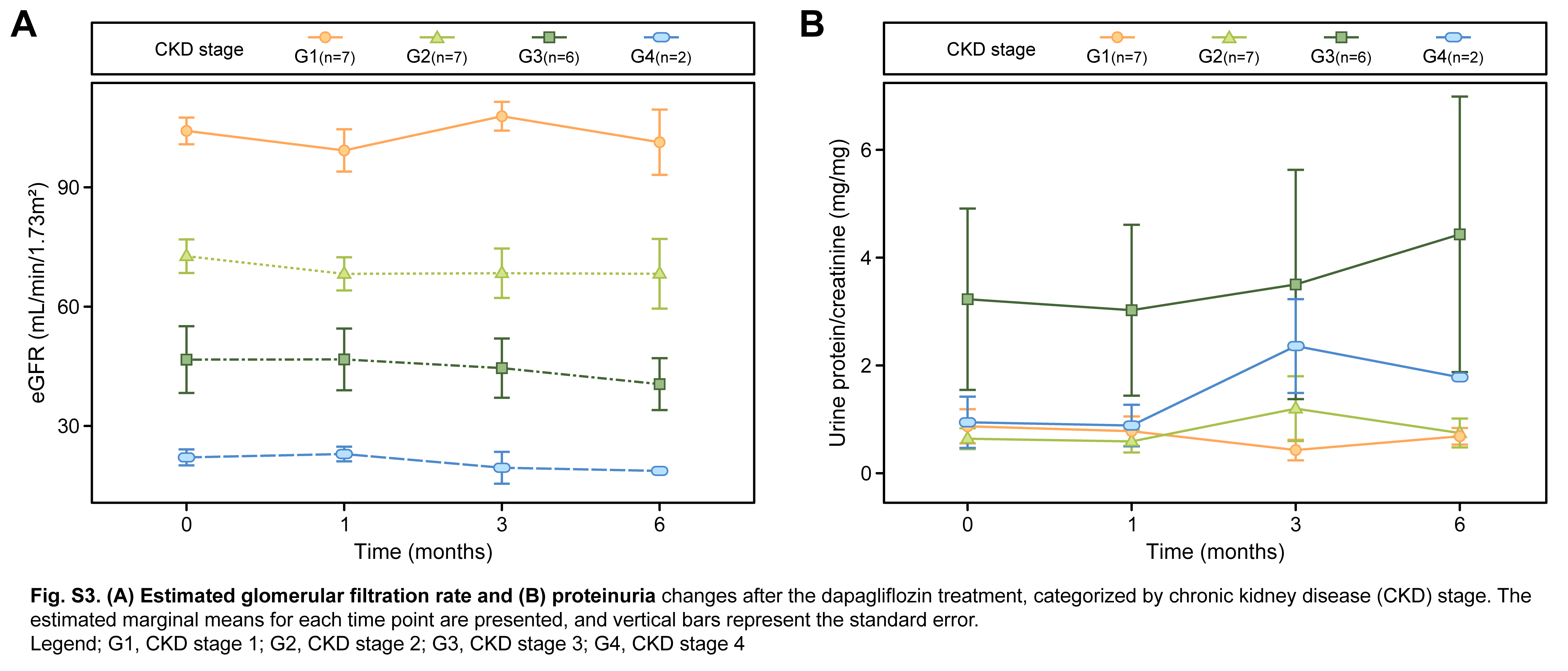

Supplement: Supplementary file 5 — Supplementary Material 4. [file 467_2024_6481_MOESM5_ESM.tif]
